# Field-Based Flow Cytometry for *Ex Vivo* Characterization of Plasmodium vivax and P. falciparum Antimalarial Sensitivity

**DOI:** 10.1128/AAC.00682-13

**Published:** 2013-10

**Authors:** B. Russell, B. Malleret, R. Suwanarusk, C. Anthony, S. Kanlaya, Y. L. Lau, C. J. Woodrow, F. Nosten, L. Renia

**Affiliations:** Department of Microbiology, Yong Loo Lin School of Medicine, National University of Singapore, National University Health System, Singaporea; Department of Parasitology, Faculty of Medicine, University of Malaya, Kuala Lumpur, Malaysiab; Laboratory of Malaria Immunobiology, Singapore Immunology Network, Biopolis, Agency for Science Technology and Research, Singaporec; Shoklo Malaria Research Unit, Mae Sot, Tak Province, Thailandd; Mahidol-Oxford-University Research Unit, Bangkok, Thailande; Centre for Tropical Medicine, University of Oxford, Churchill Hospital, Oxford, United Kingdomf

## Abstract

*Ex vivo* antimalarial sensitivity testing in human malaria parasites has largely depended on microscopic determination of schizont maturation. While this microscopic method is sensitive, it suffers from poor precision and is laborious. The recent development of portable, low-cost cytometers has allowed us to develop and validate a simple, field-optimized protocol using SYBR green and dihydroethidium for the accurate and objective determination of antimalarial drug sensitivity in freshly isolated Plasmodium vivax and Plasmodium falciparum.

## TEXT

Microscopic examination of *ex vivo* matured malaria parasites remains the gold standard method used to determine the intrinsic sensitivity of fresh Plasmodium vivax and Plasmodium falciparum isolates to antimalarial drugs (1–8). *Ex vivo* studies involve the manipulation of primary clinical samples of Plasmodium spp. in an artificial environment for no longer than 48 h. The modified WHO microtest assay is sensitive, relatively simple, and inexpensive and continues to be applied to a range of studies (9–17), especially those seeking novel antimalarial therapeutics against drug-resistant malaria (18–21). However, the microscopic examination of Giemsa-stained thick films central to this method is tedious and time-consuming and requires skilled microscopists. Moreover, large inter- and intraobserver variations of parasite staging are frequently observed (7). Attempts to find an alternative *ex vivo* method suitable for both P. vivax and P. falciparum have been largely unsuccessful due to the high background noise present in clinical isolates (caused by a number of factors, including leukocytes, red blood cell autofluorescence, gametocytes, and contaminating protein signatures in host plasma) compared with the low target signal of the maturing parasite (clinical isolates frequently have parasitemias of <0.1%) (22–24). Perhaps the most objective and direct method to determine schizont maturation is the use of flow cytometry (25–28). However, the high expense and fragility of most flow cytometers significantly limit their use in field laboratories. Fortunately, the recent development of relatively cheap, portable 2-laser flow cytometers (such as the Accuri C6; Becton, Dickinson) for the first time allows flow cytometric evaluation of *ex vivo* susceptibility assays in areas where malaria is endemic (29). Capitalizing on this new capability, we have developed a precise, accurate, fast, and simple flow cytometry (FC) method to conduct *ex vivo* drug sensitivity assays of P. vivax and P. falciparum under field conditions using only 2 colors.

Forty-eight isolates of P. vivax and 15 isolates of P. falciparum with parasitemias of between 0.02% and 0.5%, predominantly at the early ring stage (>80% of the total stages present), were collected from patients attending clinics at the Thai-Myanmar border (collected under the approved ethics protocol FMT-019-10 [Mahidol University, Faculty of Tropical Medicine Internal Review Board]). The isolates were transported to the Shoklo Malaria Research Unit (SMRU) field laboratory within 6 h of collection; the stages of parasitemia were assessed, and samples were then depleted of white blood cells (WBCs) by cellulose medium fiber (Sigma catalogue no. C6288) filtration as previously described (30) and cultured in the presence of 8 to 514 ng/ml of chloroquine diphosphate (molecular weight [MW], 515.9) (CQ) or 0.3 to 19 ng/ml sodium artesunate (MW, 406.4) (AS) using the protocol described by Russell et al. (8). At harvest (∼42 h postculture), the 200 μl of blood medium in each well was mixed, and 20 μl from each well was dispensed into a small curved-bottom tube (Micronic) and stained with 2 μl of dihydroethidium (Sigma) and 5 μl of SYBR green (made up with 63 μl of phosphate-buffered saline [PBS]) (Sigma) and incubated for 20 min at room temperature. During the staining time, thick films (3 μl packed red blood cells [RBCs]) were made from each of the wells for Giemsa staining and microscopic examination. The fluorescent staining reaction was stopped after 20 min with the addition of 400 μl of PBS, and the reaction products were stored on an ice brick until FC analysis. The FC analysis was conducted using an Accuri C6 (Becton, Dickinson), and the gating strategy was per the method of Malleret et al. (29) (see Fig. 1A in the supplemental material). However, two special modifications were made to this protocol. First, only 60,000 events rather than 300,000 events were counted (reducing the count time per well from ∼1.2 min to ∼15 s). Note that for parasitemias less than 0.1%, we suggest using 100,000 events (see Fig. 1B and C in the supplemental material). Second, no CD45 staining was necessary, as >98% of the WBCs were removed from the isolates by cellulose. Slide counts for the microscopy were conducted as described by Russell et al. (8). The proportion of events in the target gate (for cytometry) or the mature schizonts (for microscopy) at each of the treatments was normalized to that in the drug-free control. The proportion of schizont maturation at each corresponding drug concentration was then entered into the online ICEstimator (http://www.antimalarial-icestimator.net/MethodIntro.htm), and the 50% inhibitory concentration (IC_50_) was calculated by nonlinear regression analysis (31, 32).

**Fig 1:**
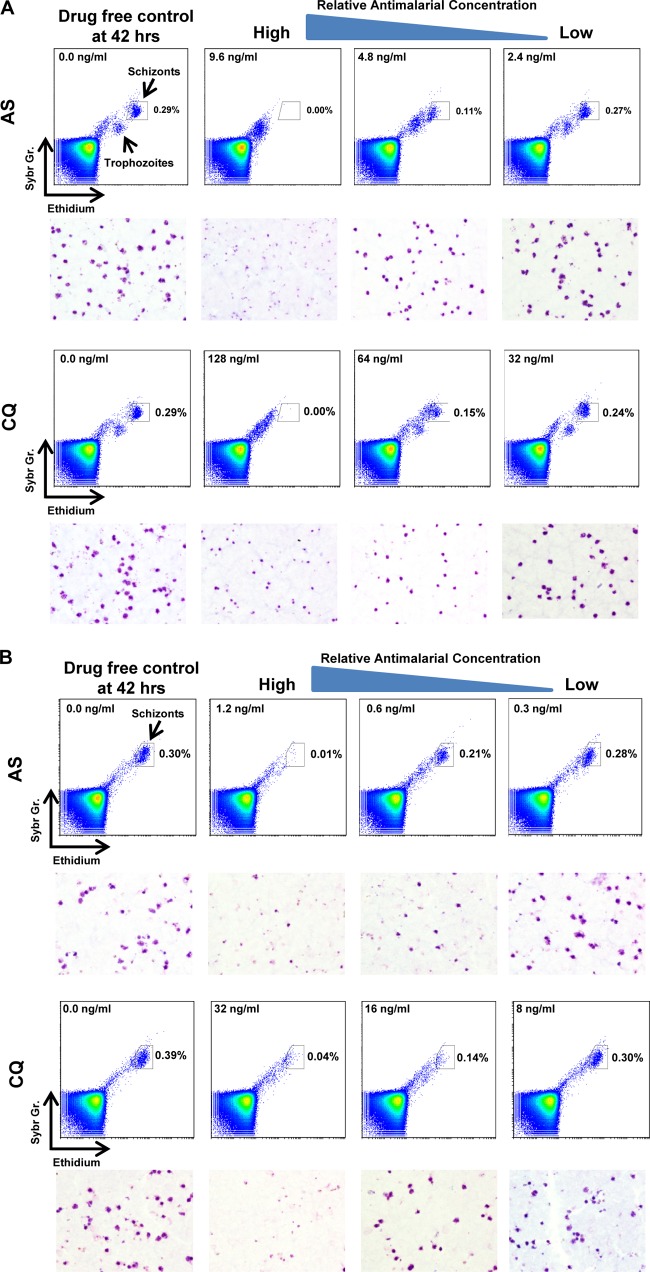
Representative flow cytometry plot outputs from chloroquine (CQ) and artesunate (AS) sensitivity assays conducted on P. falciparum (A) and P. vivax (B). The target gate representing schizont development events is indicated on each plot. Underneath the plots are the corresponding micrographs of Giemsa-stained thick films collected from the same culture wells.

After 42 h of culture in the drug-free controls, the schizont “target gate” on the cytometer plot corresponding to the cluster of events with the highest levels of DNA (SYBR green, *y* axis) and RNA (dihydroethidium, *x* axis) can be clearly discerned for both species (Fig. 1), with the number of events in the FC plot target gate corresponding to the presence or absence of schizonts in the thick films (Fig. 1). As it is important to ensure that later-stage parasites and gametocytes initially present in the precultured isolate (time 0) do not confound the events in the schizont gate postculture, we ran an FC analysis on these initial samples. Any background events present near the target gate were then later subtracted from the events present at gates of the control and treatments postculture.

The culture success rate for both species was good, with 95.8% (46/50) and 86.7% (13/15) of P. vivax and P. falciparum samples, respectively, reaching at least 60% schizonts in the drug-free control after at least 42 h culture. Of the 46 successful P. vivax cultures, we were unable to model the IC_50_ data for one of the CQ assays.

**Fig 2:**
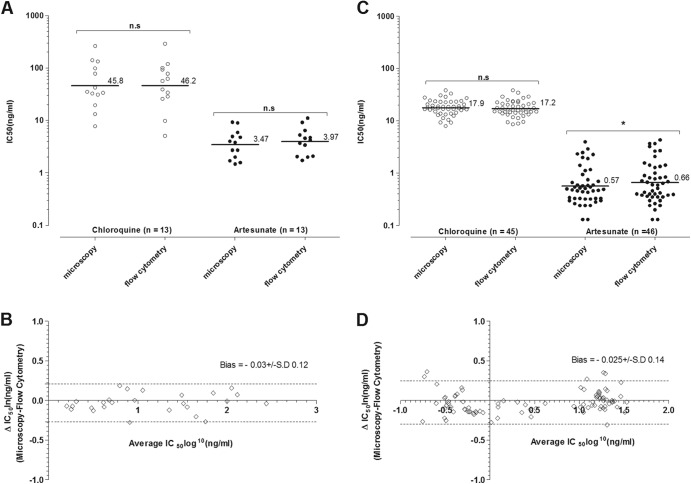
*Ex vivo* sensitivities of Plasmodium falciparum (A) and Plasmodium vivax (C) to chloroquine (CQ) and artesunate (AS), compared using microscopy and flow cytometry (Accuri C6). Solid horizontal lines and associated values are the geometric mean IC_50_ (ng/ml). A paired *t* test showed that there was a significant difference (*P* < 0.01) between the AS and FC IC_50_s, as calculated by microscopy. Bland-Altman comparisons of IC_50_s for P. falciparum (B) and P. vivax (D) (AS and CQ combined) were determined by microscopy and flow cytometry. The upper and lower 95% limits of agreement are denoted by the dotted lines.

The geometric mean IC_50_s of P. vivax CQ and AS determined by microscopy and FC were 17.93 ng/ml (95% CI, 16.2 to 19.84; *n* = 45) versus 17.20 ng/ml (95% CI, 15.52 to 19.07; *n* = 46) and 0.57 ng/ml (95% CI, 0.45 to 0.72; *n* = 45) versus 0.66 ng/ml (95% CI, 0.51 to 0.86; *n* = 45), respectively (Fig. 2C). For P. falciparum, the geometric mean IC_50_s of CQ and AS determined by microscopy and FC were 45.82 ng/ml (95% CI, 24.22 to 84.2; *n* = 13) versus 46.22 ng/ml (95% CI, 24.22 to 88.2; *n* = 13) and 3.47 ng/ml (95% CI, 2.38 to 5.1; *n* = 13) versus 3.97 ng/ml (95% CI, 2.78 to 5.67; *n* = 13) (Fig. 2A). Paired *t* test analysis showed that the only comparison where there was a significant difference was the sensitivity of P. vivax to AS (Fig. 1C) (*P* < 0.01). It should be noted that the interspecies differences between the IC_50_s for CQ and AS are expected and already noted in numerous studies; however, the mechanism behind this still remains unknown.

Bland-Altman analysis indicated good agreement between the methodologies (independent of drug type used) (Fig. 2B and D). There was a slight bias toward higher IC_50_s with the flow cytometry method for both P. falciparum (−0.03 log_10_ units) and P. vivax (−0.025 log_10_ units).

In summary, the antimalarial sensitivity data for the new FC assay matched those of the traditional microscopy very closely. In the one case where there was a significant difference between the IC_50_ analysis of FC and microscopy, the actual mean difference in AS IC_50_ for P. vivax was less than 0.1 ng/ml, which is unlikely to be of biological significance. This 0.1-ng/ml disparity should also be put in the context of interreader variability between microscopists, which in our experience can be an order of magnitude greater. It should also be noted that the time to acquire data from the FC method is only ∼2 min per drug (8 wells), compared to 18 min by microscopy. While assay described here used an extended exposure of AS (42 h), we have also used a more physiological 2-h “pulse exposure” of AS at the beginning of the FC assay to mimic the <1-h half-life pharmacokinetic profile of this drug *in vivo* (this results in an ∼10-fold increase in the AS IC_50_[data not presented]). In conclusion, our data support the use of this simple FC protocol as a precise and more objective alternative to the microscopic determination of antimalarial drug sensitivity in fresh isolates of P. vivax and P. falciparum. Further studies involving a wider range of drugs are planned.

## Supplementary Material

Supplemental material
