# Supplementary material for: Field-Based Flow Cytometry for Ex Vivo Characterization of Plasmodium vivax and P. falciparum Antimalarial Sensitivity
Source: Antimicrob Agents Chemother. 2013 Oct;57(10):5170–4. doi: 10.1128/AAC.00682-13 (PMC3811473; doi:10.1128/AAC.00682-13)
Supplement: Supplemental material [file supp_57_10_5170__index.html]

Supplemental material 

# Field-Based Flow Cytometry for *Ex Vivo* Characterization of Plasmodium vivax and P. falciparum Antimalarial Sensitivity

## Supplemental material

**Files in this Data Supplement:**

- Supplemental file 1 -

  Flow cytometry gating strategy for schizont parasitemia determination from 100,000 events collected from a 42-h *ex vivo* culture of *P. vivax*.

  PDF, 561K
