# Supplementary material for: Field-Based Flow Cytometry for Ex Vivo Characterization of Plasmodium vivax and P. falciparum Antimalarial Sensitivity
Source: Antimicrob Agents Chemother. 2013 Oct;57(10):5170–4. doi: 10.1128/AAC.00682-13 (PMC3811473; doi:10.1128/AAC.00682-13)
Supplement: Supplemental material [file AAC.00682-13_zac010132209so1.pdf]

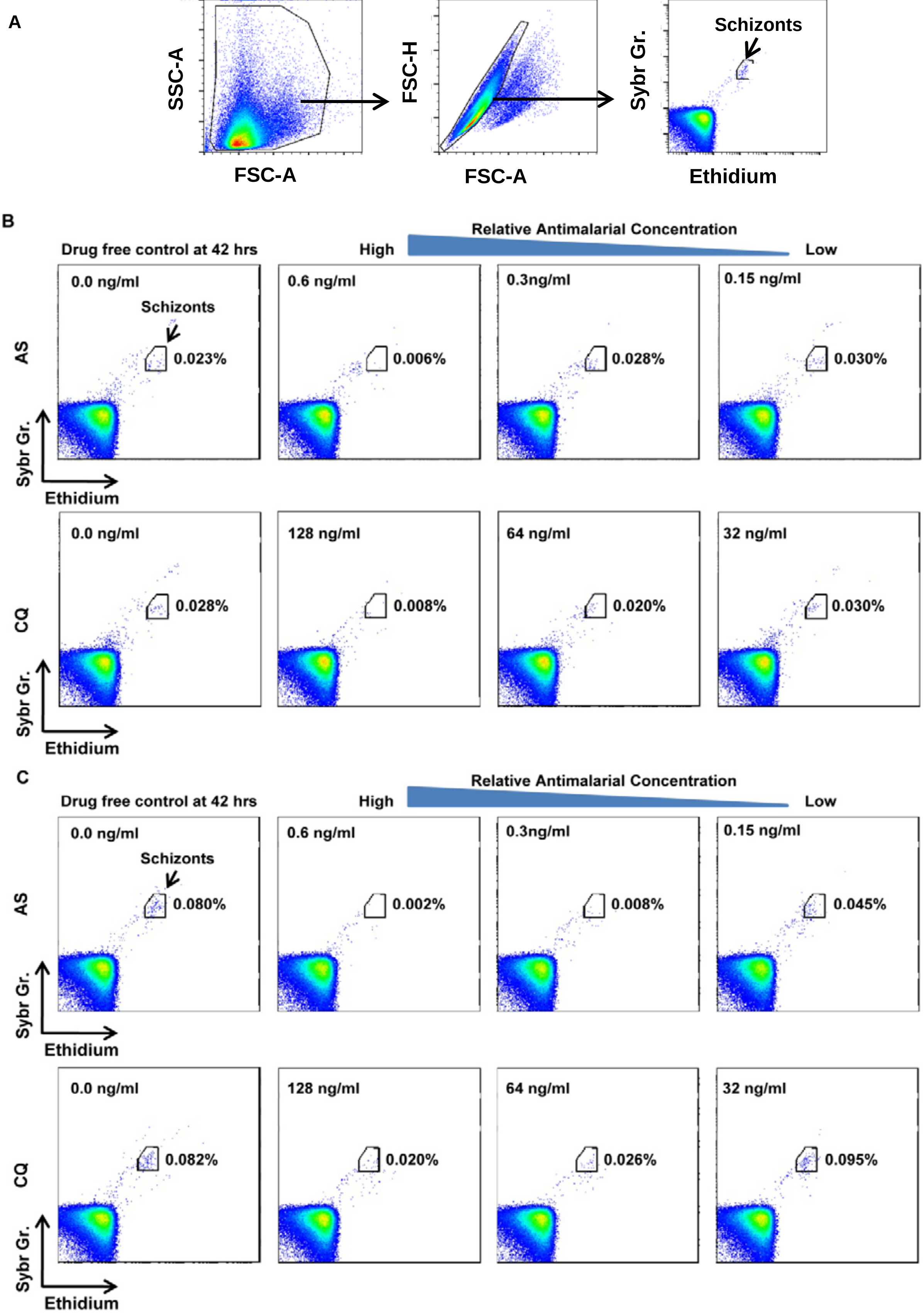

Supplementary Figure 1: Flow cytometry gating strategy for schizont parasitemia determination from 100, 000 events collected from a 42hr ex vivo culture of *P.vivax* (A).The first panel on the left gates only cellular events using Side Scatter Area (SSC-A) and Forward Scatter Area(FSC-A) , the second panel shows the exclusion of cells stuck together using Forward Scatter Height (FSC-H) and FSC-A. The final panel in this series shows the gating of the schizonts using Sybr Green and Dihydroethidium. Representative flow cytometry plot outputs from chloroquine (CQ) and artesunate (AS) sensitivity assays conducted on (B) *P. falciparum* and (C) *P. vivax* isolates with relatively low parasitemas (Less than 0.1% parasitemia)
